# Mediating Water Temperature Increases Due to Livestock and Global Change in High Elevation Meadow Streams of the Golden Trout Wilderness

**DOI:** 10.1371/journal.pone.0142426

**Published:** 2015-11-13

**Authors:** Sébastien Nusslé, Kathleen R. Matthews, Stephanie M. Carlson

**Affiliations:** 1 Department of Environmental Science, Policy & Management, University of California, Berkeley, California, United States of America; 2 Forest Service Pacific Southwest Research Station, United States Department of Agriculture, Albany, California, United States of America; Aberystwyth University, UNITED KINGDOM

## Abstract

Rising temperatures due to climate change are pushing the thermal limits of many species, but how climate warming interacts with other anthropogenic disturbances such as land use remains poorly understood. To understand the interactive effects of climate warming and livestock grazing on water temperature in three high elevation meadow streams in the Golden Trout Wilderness, California, we measured riparian vegetation and monitored water temperature in three meadow streams between 2008 and 2013, including two “resting” meadows and one meadow that is partially grazed. All three meadows have been subject to grazing by cattle and sheep since the 1800s and their streams are home to the imperiled California golden trout (*Oncorhynchus mykiss aguabonita*). In 1991, a livestock exclosure was constructed in one of the meadows (Mulkey), leaving a portion of stream ungrazed to minimize the negative effects of cattle. In 2001, cattle were removed completely from two other meadows (Big Whitney and Ramshaw), which have been in a “resting” state since that time. Inside the livestock exclosure in Mulkey, we found that riverbank vegetation was both larger and denser than outside the exclosure where cattle were present, resulting in more shaded waters and cooler maximal temperatures inside the exclosure. In addition, between meadows comparisons showed that water temperatures were cooler in the ungrazed meadows compared to the grazed area in the partially grazed meadow. Finally, we found that predicted temperatures under different global warming scenarios were likely to be higher in presence of livestock grazing. Our results highlight that land use can interact with climate change to worsen the local thermal conditions for taxa on the edge and that protecting riparian vegetation is likely to increase the resiliency of these ecosystems to climate change.

## Introduction

It is now widely acknowledged that contemporary climate change will severely impact most ecosystems of the planet [1]. One major consequence of recent climate change is that global temperatures have increased approximately 0.85°C on average since the end of the 19^th^ century, and that extreme climatic events are increasing in frequency, including storms, floods, droughts, and heat waves [1,2]. Freshwaters ecosystems are not immune to these changes and modified hydrology and thermal regimes will alter the quality of habitat for sensitive biota [3]. As an example, rivers in the U.S.A. have already experienced warming of about 0.2°C per decade since 1990, with the highest increases reported in urbanized areas [4].

Organisms can respond to these changes by moving to areas of suitable conditions if there is enough connectivity and migration potential, adapting to new (changed) conditions [5], being extirpated from unsuitable habitat, or going extinct. Species in mountainous ecosystems are particularly sensitive to climate change as their suitable habitats are being shifted upwards toward the summit and compressed with nowhere else to go. While most research has focused on birds and mammals in high elevation ecosystems [6–8], fishes face similar challenges and may need to move upstream to find cooler conditions but, again, this is not always possible [9,10], particularly when they are confined to isolated lakes or already occur in uppermost, headwater habitats. Warming temperatures in high-elevation rivers may create challenges for cold-water fishes that rely on cold, highly oxygenated, water to complete their life cycle [11,12].

Beyond the direct impacts of climate, anthropogenic stressors such as habitat modification and pollution may interact with global change to amplify its impact [13]. For example, the collapse of many fisheries has been attributed to the combined effect of global change, overfishing, and pollution [14]. Similarly, the combination of climate-induced drought and land use such as deforestation or intensive agriculture has triggered dramatic changes in forest communities and shifts toward drier biomes [15]. One form of land use that may be interacting with climate change to exacerbate risks for sensitive species in high elevation montane ecosystems is cattle grazing. In the arid western USA, the practice of livestock grazing on public lands is widespread, despite many demonstrated negative effects on biodiversity [16,17]. For example, high elevation montane meadows in the Sierra Nevada mountain range have been used for livestock grazing since the 1800s, which has led to degradation of streams and adjacent riparian zones [18–21]. Beyond the direct effects of grazing, cattle usually concentrate around water sources to drink, trampling vegetation and stream banks, which can result in stream bank erosion and channel incision [17,22–24]. Cattle activities also compact the soil, which limits water availability to vegetation [25] and, in dry areas, increases xerification [23]. Concerns about the interactive effects of cattle grazing on public lands and climate change led the President of the American Fisheries Society, Prof. Robert Hughes, to call for a great reduction of grazing on public lands [26].

In this study, we investigated the effect of livestock on stream water temperature in high elevation meadows of the Golden Trout Wilderness, a protected area in the Sierra Nevada, California, USA. These high elevation wetland ecosystems provide water regulation services [18,27] and harbor unique biodiversity, including the California State fish: the California golden trout (*Oncorhynchus mykiss aguabonita*). The removal of vegetation and the degradation of the riparian zone due to livestock activities are particularly deleterious for cold-water salmonids in the western USA [28], especially the native golden trout, a species already at risk due to degraded habitat, genetic introgression, limited distribution, competition with exotic species, and more recently, rising water temperatures in this region [19,29]. This species could be particularly sensitive to even more reduction in their suitable range due to warming because of their already restricted distribution in headwater meadow streams [29–31]. We compared three meadow systems under different grazing management, including two meadows where cattle have been excluded since 2001 and a third meadow where an experimental cattle-exclusion area was constructed in 1991. In the partially grazed meadow, we examined the direct effect of cattle on the vegetative cover and stream shading inside (i.e., the ungrazed area) and outside (i.e., the grazed area) the cattle exclosure, and we measured temperature along the stream in both areas. Additionally, we compared water temperatures among meadows using temperature data collected over six years. Together, these analyses allowed us to assess the influence of cattle on stream temperatures in these meadow streams. Finally, we modeled expected future temperatures under different climate change scenarios to understand how these two human impacts interact to influence the water temperature of golden trout stream habitat.

## Methods

### Study area

The study was conducted in the Golden Trout Wilderness, under the Inyo National Forest Special Use Permit (LVD080003P), issued by USDA Forest Service. The Golden Trout Wilderness is at the southern end of the Sierra Nevada, California (118°15'N, 36°22'W) and is characterized by large subalpine meadows characterized by elevations higher than 500 meters, shallow ground water, fine textured superficial soils, and the dominance of herbaceous vegetation [23,32]. Meadow vegetation is typically dominated by sagebrush (*Artemisia cana*), while riparian vegetation consists mostly of sedge (*Carex spp*.) and willow (*Salix spp*.). The California golden trout dominates the fish fauna of these meadow systems, and a second native fish species, the Sacramento sucker (*Catostomus occidentalis*), is also present in the South Fork Kern River, but is rarely observed [19].

### Cattle exclusion

**Fig 1:**
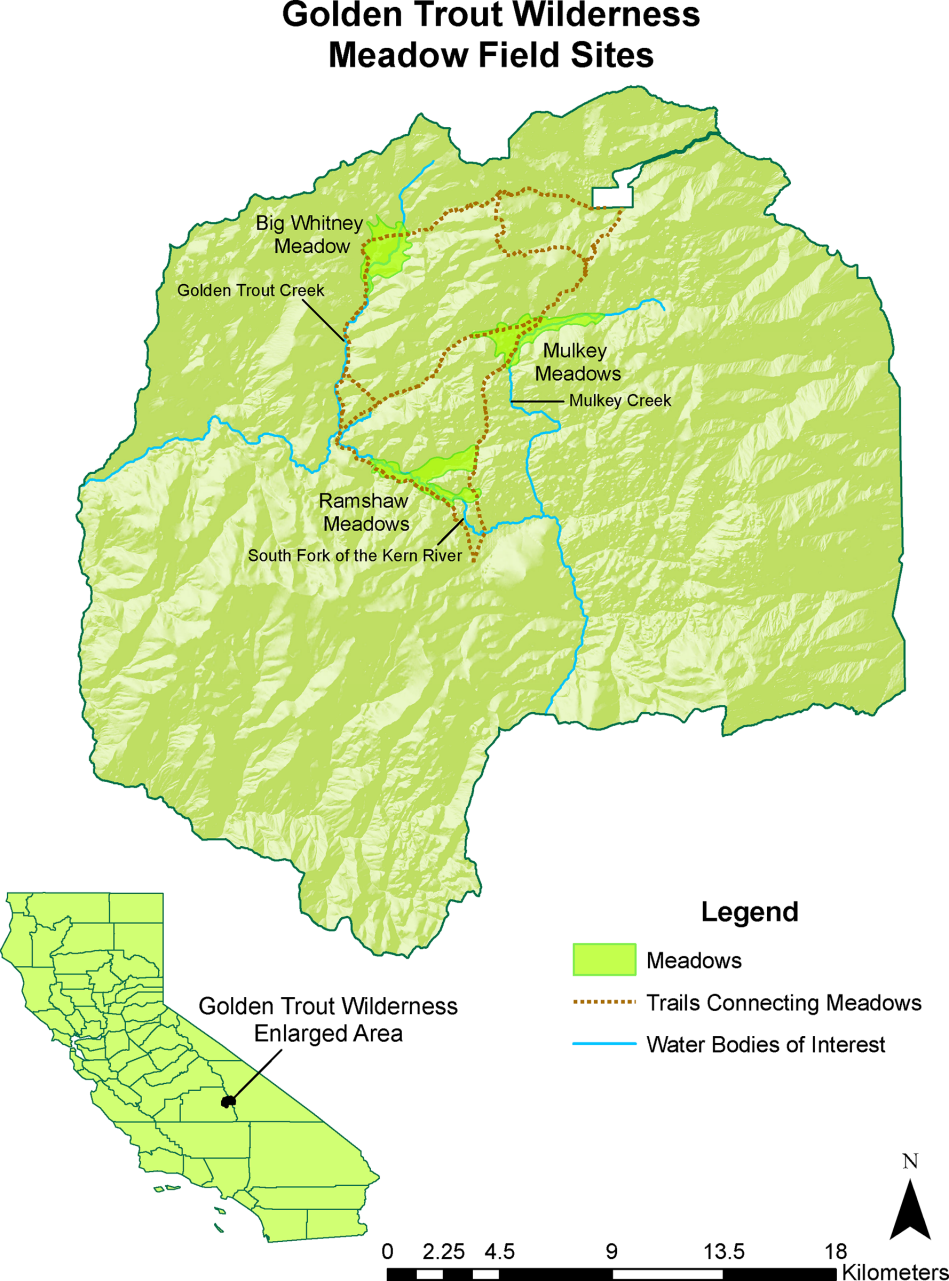
Study area. Data were collected in three distinct meadow systems of the Golden Trout Wilderness, California, a protected area within the Inyo National Forest in the Sierra Nevada mountains, which is the last remaining habitat of the Golden Trout (*Oncorynchus mykiss aquabonita*). Water temperature was measured between 2008–2013 in three rivers: (1) Mulkey Creek, within Mulkey Meadows, between 2827–2844 m in elevation, (2) the South Fork of the Kern River within Ramshaw Meadows, between 2629–2648 m, and (3) the Golden Trout Creek within Big Whitney Meadow, between 2931–2964 m.

To protect the meadows from damage linked to grazing, the Inyo National Forest has removed cattle from some meadows and constructed cattle exclosures in several other meadows along the river channel [19]. We investigated three large meadows systems (5–7 km long), grazed by cattle and sheep since the 1800s, in the largest meadow complex in the Sierra Nevada occurring in depositional basins of the Kern Plateau (Fig 1, Table 1): (1) Mulkey Meadows (36°24’19”N, 118°11’42.14”W, elevation: 2838 m), where cattle are partially excluded by a cattle exclosure that was constructed in 1991, and two other meadows where cattle have been excluded completely since 2001: (2) Ramshaw Meadows (36°20’53”N, 118°14’52.62”W, elevation: 2640 m) and (3) Big Whitney Meadow (36°26’23”N, 118°16’11.66”W, elevation: 2963 m). These meadows are generally covered with snow from November to May, and are located in a semi-arid region where annual precipitation is 50–70 cm and mostly in the form of snow [19].

**Table 1:** Meadows information.

Meadow (area)	Elevation		Average velocity	Slope within study area	Watershed	
	average	(min—max)			area	(mean / max elevation)
Ramshaw	2638 m	(2629 m—2648 m)	0.21 ± 0.17 m/s	1.12 m/100m	77.5 Km2	(2858 m / 3505 m)
Mulkey grazed (outside exclosure)	2840 m	(2836 m—2844 m)	0.13 ± 0.13 m/s	1.04 m/100m	105.5 Km^2^	(2878 m / 3535 m)
Mulkey ungrazed (within exclosure)	2833 m	(2827 m—2839 m)	0.10 ± 0.11 m/s	0.55 m/100m	105.5 Km^2^	(2878 m / 3535 m)
Big Whitney	2948 m	(2931 m—2964 m)	0.35 ± 0.18 m/s	1.78 m/100m	154.3 Km^2^	(2978 m / 3927 m)

### Environmental and temperature data

In 2014, in Mulkey Meadows, we walked the river inside the study area and measured all willows within 2 meters of the bank with a measuring rod: the height (cm) and the GPS location of each willow were recorded. To characterize water temperatures in the meadows, we deployed temperature probes (Onset HOBO Water Temp Pro v2 ± 0.21°C and tidbits ± 0.2°C) throughout the stream study sites. The probes logged temperatures for 3–6 years between 2008–2013 at each of the three sites, but only the data for the three overlapping years, 2010–2012, were used for our ‘among meadow’ comparisons. We used data from 30 probes in Mulkey (13 probes in the ungrazed area [i.e., inside the cattle-exclosure] and 17 probes from the grazed area [i.e., outside the cattle-exclosure]), 21 in Big Whitney, and 30 in Ramshaw. Probe temperatures were periodically checked and compared to the YSI 55 DO and temperature meter to ensure accuracy. Precise GPS coordinates, depth of the probe, flow, vegetation type (willow dominated, sedge dominated, grass dominated, or without vegetation), and habitat characteristics (pool or riffle, and in Mulkey whether it was outside or inside the cattle-exclosure) were recorded when probes were deployed. Additionally, at each probe, solar radiation was measured in 2013 using a Solmetric Suneye 210, a handheld recording device measuring standardized solar radiation by considering latitude, solar azimuth, time of day, and date while integrating local features including channel aspect, topography, and streamside vegetation. To simplify the analyses and perform autoregressive models, solar radiation was transformed into a binary variable, being 1 when solar exposure was almost total (solar exposure ≥ 98%) or 0 when shade was present (solar exposure < 98%). Tributaries to each meadow stream were reported from maps. Watershed areas and elevation were calculated in ArcGIS from USGS HUC12 level watershed boundaries and USGS 30m National Elevation Dataset.

### Water temperature metrics

For the sake of simplicity, we focus on the effect of high temperatures during summer. Therefore we used a subset of the temperature dataset that included the eight weeks each year that experienced the highest temperature (from the 24th to the 31st week of the year, i.e., mid-June to early August). Prior to analyses, we removed anomalous data as some probes were out of the water during a certain period; when this happened (on 15 occasions), the whole daily record of the probe was discarded. To summarize the tremendous amount of individual data (3,446,765 individual temperature records) we constructed three summary metrics: for each probe, we computed the daily average temperature (DavgT), the daily maximum temperatures (DmaxT) and the daily minimal temperature (DminT). We then computed the median daily values over seven day moving windows for those metrics (Table 2), i.e., the weekly average temperature (WavgT), the weekly maximal temperature (WmaxT), the weekly minimal temperature (WminT). We focused on the median, instead of the mean, so that any aberrant or exceptional values did not have a strong influence on results. Finally, we computed the maximum weekly average temperature MWavgT, and the maximum weekly maximum temperature MWmaxT, which are common measures found in the literature [33] and can be understood as the average and maximum values, respectively, during the warmest consecutive seven days of measurements. These values can be compared to measures of chronic (i.e., sub-lethal) temperature exposure for MWavgT and acute (i.e., lethal) temperature exposure for MWmaxT [34] to understand the consequences for the focal organism. See S1 Fig for an example of how these temperature metrics were calculated, using data collected over three weeks as an example.

**Table 2:** Temperature summary.

Meadow (area)	Weekly minimum temperature (WminT)	Weekly average temperature (WavgT)	Weekly maximum temperature (WmaxT)
Ramshaw	8.2 ± 1.5°C	11.8 ± 1.3°C	16.1 ± 2.7°C
Mulkey grazed (outside exclosure)	8.6 ± 1.7°C	13.2 ± 1.4°C	18.6 ± 3.2°C
Mulkey ungrazed (within exclosure)	8.3 ± 1.4°C	13.0 ± 1.5°C	18.4 ± 3.2°C
Big Whitney	6.6 ± 1.1°C	10.7 ± 0.4°C	16.5 ± 1.8°C

### Spatial and temporal autocorrelation

A major issue with thermal datasets is autocorrelation that may lead to erroneous conclusions if not taken into account. The presence of autocorrelation does not change the magnitude of the differences observed (the regression coefficients are unbiased), but the variability of these coefficient is underestimated, which inflate t and F statistics and result in artificially narrow confidence intervals [35]. In other words, the effect size is the same, but the associated *p*-value differs when autocorrelation is present. Consequently, when dealing with autocorrelation there is a trade-off between accounting for autocorrelation and decreasing the power of analyses by reducing the number of observations.

There are two major sources of autocorrelation within our dataset: (1) *temporal autocorrelation* with measurements at a given probe recorded every 20–30 minutes that are not independent of one another (temperature at a particular time is likely to be correlated with the temperature one hour, day, or week before) and (2) *spatial autocorrelation* between probes (the location of individual probes were distributed along a longitudinal transect, therefore the measures at one probe location are likely to be correlated with values from upstream probes). There is also a third issue, which is *repeated measurements* of the same object (each probe is measured many times during the course of the study).

To account for temporal autocorrelation, we compared values averaged over time, including yearly, monthly, or weekly averages of the temperature metrics (WminT, WavgT, and WmaxT). We tested temporal autocorrelation with autoregressive models, that is, we calculated the correlation between the focal temperature metric at time *t* and time *t+1* (*t* in weeks, months, or years). We found that weekly estimates were significantly autocorrelated for WavgT (*p <* 0.05), but not for WminT or WmaxT (*p >* 0.05). We found no evidence of temporal autocorrelation for the monthly or yearly estimates (all *p >* 0). Since our results illustrate the trade-off between accounting for temporal autocorrelation and decreasing the power of analyses, we report the values and statistics for all three timescales (week, month, and year) in Table 3.

**Table 3:** Temperature differences.

Meadows	Averaged over	Weekly minimum temperature (WminT)		Weekly average temperature (WavgT)		Weekly maximum temperature (WmaxT)	
Mulkey (grazed)	year	-0.03 ± 0.36°C	*t*_6.0_ = 0.08 [NS]	1.63 ± 0.48°C	*t*_6.0_ = 3.4 [**]	3.53 ± 1.19°C	*t*_8.0_ = 2.96 [*]
vs,	month	-0.02 ± 0.23°C	*t*_22.9_ = 0.1 [NS]	1.65 ± 0.26°C	*t*_22.9_ = 6.24 [***]	3.53 ± 0.61°C	*t*_31.0_ = 5.77 [***]
Ramshaw	week	-0.03 ± 0.14°C	*t*_62.8_ = 0.18 [NS]	1.64 ± 0.17°C	*t*_62.2_ = 9.42 [***]	3.54 ± 0.41°C	*t*_58.9_ = 8.64 [***]
Mulkey (grazed)	year	0.28 ± 0.36°C	*t*_6.0_ = 0.78 [NS]	0.24 ± 0.48°C	*t*_6.0_ = 0.5 [NS]	0.22 ± 1.19°C	*t*_8.0_ = 0.18 [NS]
vs.	month	0.31 ± 0.23°C	*t*_22.9_ = 1.35 [NS]	0.31 ± 0.26°C	*t*_22.9_ = 1.17 [NS]	0.29 ± 0.61°C	*t*_31.0_ = 0.47 [NS]
Mulkey (ungrazed)	week	0.31 ± 0.14°C	*t*_62.8_ = 2.24 [*]	0.26 ± 0.17°C	*t*_62.2_ = 1.49 [NS]	0.19 ± 0.41°C	*t*_58.9_ = 0.47 [NS]
Mulkey grazed	year	1.59 ± 0.36°C	*t*_6.0_ = 4.48 [***]	2.54 ± 0.48°C	*t*_6.0_ = 5.28 [***]	2.62 ± 1.19°C	*t*_8.0_ = 2.20 [.]
vs.	month	1.79 ± 0.24°C	*t*_23.0_ = 7.36 [***]	2.65 ± 0.27°C	*t*_23.1_ = 9.65 [***]	2.63 ± 0.63°C	*t*_31.0_ = 4.17 [***]
Big Whitney	week	1.76 ± 0.15°C	*t*_63.2_ = 11.43 [***]	2.52 ± 0.19°C	*t*_63.2_ = 13.18 [***]	2.54 ± 0.44°C	*t*_63.7_ = 5.72 [***]

[NS] = *p*-value > 0.05

[*] = *p*-value < 0.05

[**] = *p*-value < 0.01

[***] = *p*-value < 0.001

To account for spatial of autocorrelation, we included the position of the probe in the stream, i.e., the probe location, as a covariate in our models, and when necessary, we used autoregressive models (see below). Finally, we used mixed-models with the individual probe ID as a random effect so that repeated measurements from the same probe were taken into account. To detect and quantify autocorrelation, we used Moran’s *I* autocorrelation coefficient on the residuals of our models, which calculates the correlation between neighboring data weighted by the distance between pairs of data points [36]. For temporal autocorrelation, the distance is a temporal distance (in days, weeks or months between two measurements).

### Environmental data within a partially grazed meadow, Mulkey Meadows

We tested for evidence of spatial autocorrelation within the environmental data (vegetation type, willow height, distance between consecutive willows, solar exposure, and flow) with Moran’s *I* coefficient. Specifically, we calculated autocorrelation coefficients on the residuals of the linear models with the environmental variable included as the response variable and the exclosure (0 = inside exclosure/ungrazed ungrazed, 1 = outside exclosure/grazed) as the explanatory variable. For willow height, distance between consecutive willows, and flow, we used linear models. For vegetation type and solar exposure, we instead used logistic regression. For spatial autocorrelation measurements, vegetation type was transformed into a binary variable: dominated by willow (1) or not (0). When spatial autocorrelation was detected, we compared the environmental factors inside and outside the exclosure with autoregressive models that included longitude, latitude, and their squared values as covariates. As autocorrelation only inflates the degrees of freedom without changing the coefficients, we did not perform a correction for the variables that were not significantly different between the within/ungrazed and outside/grazed areas. Autocorrelation measurements were performed in R [37] with the *MoranI()* function in the *ape* package [38].

### Effects of a livestock exclosure on stream temperature

To determine the effect of livestock grazing on the maximal temperatures that can be reached across the entire site, we extracted the maximal value of weekly maximal temperature (MWmaxT). This value is the average of the maximal temperatures that can be reached each day during the warmest week in a certain location over the entire study duration. Using only one value per probe, to deal with temporal autocorrelation and repeated measurement, we then investigated—in each of the three meadows—the link between longitudinal distance and maximal temperature with linear regressions, with distance calculated as the linear distance between each probe and the most upstream probe. In Mulkey Meadows, we could not use an ANCOVA-type analysis with both treatments in the same model as the slope of the model differed between treatments (grazed and ungrazed). Instead, we performed two different models, and assessed the temperature trend independently inside (ungrazed area) and outside (grazed area) the cattle exclosures. For each linear model, we checked for spatial autocorrelation with the estimated Moran’s *I* coefficients on the residuals.

### Temperature differences among meadows

To compare the weekly temperatures metrics (WminT, WavgT, and WmaxT) among the three rivers and between the two treatments in Mulkey Meadows, we calculated the monthly mean of each temperature metric to exclude most temporal autocorrelation without overly penalizing the power of the analysis. We then averaged these monthly values over the probes to remove spatial autocorrelation. In other words, we used one value per month and per river (two in Mulkey Meadows, one for each treatment [grazed/ungrazed]) for each temperature metric. As a comparison, we repeated our analyses with yearly and weekly estimates and found, as expected [35], similar values, but different levels of confidence (Table 3). Finally, we compared the means of the different temperature metrics in the three meadows with linear mixed-models, and used the probe ID to account for multiple measurements in the same probe. The averaged values were treated as the response variable, with the meadow as a fixed effect and the month of measurement included as a random effect. In other words, each month during the summer, we estimated the average weekly minimal (WminT), average (WavgT), and maximal (WmaxT) values, and then compared the differences among the three rivers, using only the data from the grazed part of Mulkey Meadows. All data analyses were performed in R [37]. The model was fit with the *lmer()* function from the package *lme4* [39] and the additional package *lmerTest()* [40]. The residuals of the models were tested for temporal autocorrelation with Moran’s *I* coefficient tests with the *MoranI()* function in the *ape* package [38].

### Future temperature scenarios

Global warming predictions include several scenarios and can be summarized as the magnitude of increase in the average air temperature [1]. Modeling the relationship between air and water temperature is complex [41], and a linear increase of 1:1°C water/air temperature increase is rarely observed [42]. We used a conservative value below the lower bound of the Morrill *et al*. estimation [42], i.e., a 0.5°C increase in water temperature for each 1°C increase in air temperature, and applied it to four different scenarios for the end of the 21^st^ century: (1) no change expected, reflecting the current situation; (2) an optimistic model with 1°C increase in air temperature / 0.5°C increase for water temperature, which corresponds to an air temperature increase between 0.3°C and 1.7°C [1]; (3) a pessimistic model with 3.7°C increase in air temperature / 1.8°C increase for water temperature, which is the average of the most pessimistic scenario predicted by the IPCC, i.e., an increase of 2.6°C to 4.8°C [1]; and (4) a cataclysmic model with 5.6°C air temperature increase / 2.8°C increase for water temperature, which represents the worst case scenario for the U.S.A. [43].

To estimate future maximal stream temperatures based on the average water temperature in each scenario for the three meadows, we used linear mixed models with the observed maximal daily temperature (DmaxT) in each probe as the response variable, and the weekly average temperature (WavgT) for each probe as a fixed effect. To account for repeated measures, we included date as a random effect. Assuming a normal distribution of the residuals of the model, we computed three confidence intervals around the predicted maximal temperature (50%, 95% and 99%) given a weekly average temperature. These interval limits represent the temperatures that might be reached every other year (50% CI), every 20 years (95% CI), and once every century (99% CI). To compare the model results among meadows, we used another model including all the data available from the three meadows, and added the meadow and its interaction with the weekly average temperature (WavgT) as additional fixed effect. To increase the precision of our models, we used all the data collected in each meadow: including data collected from 2008 to 2013 in Mulkey, from 2010 to 2013 in Big Whitney, and from 2008 to 2012 in Ramshaw. Because of the influence of the upstream probes on water temperature, there are no marked differences between the two areas (grazed/ungrazed) in Mulkey Meadows (Tables 2 and 3). We therefore choose to pool the data between the two areas of Mulkey for this analysis, which yields a conservative estimate of temperature change in this partially-grazed meadow as the ungrazed portion is hypothesized to be relatively cooler. The Mulkey “treatment” in this analysis should therefore be considered a “partially grazed” treatment. We note, however, that a parallel analysis using only the grazed part of Mulkey showed very similar results.

## Results

### Riparian vegetation and solar exposure in the grazed and ungrazed areas of Mulkey Meadows

**Fig 2:**
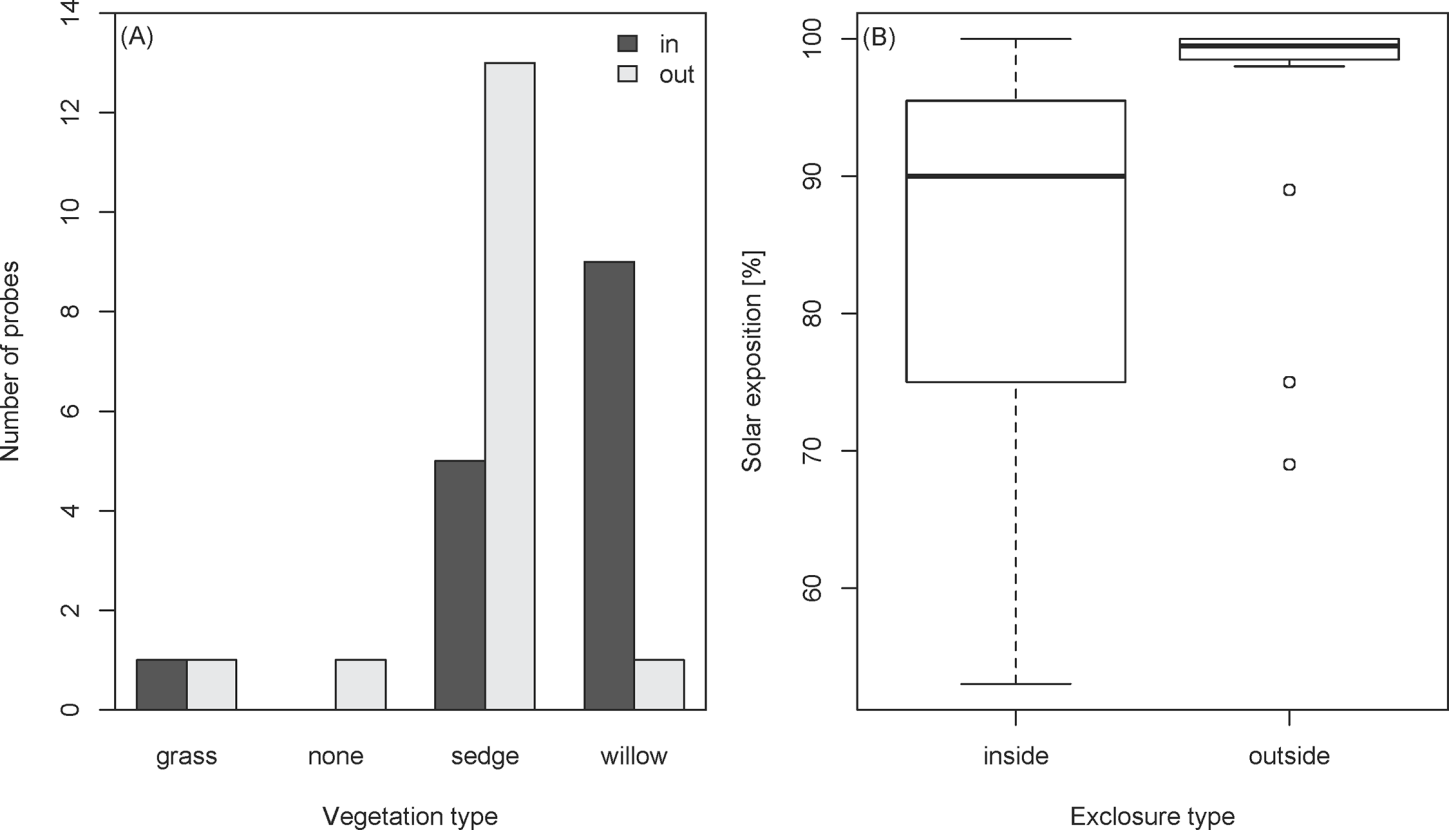
Environmental data in Mulkey. (A) Number of probes in Mulkey Meadows with the dominant type of vegetation indicated (grass dominated, no vegetation, sedge dominated, or willow dominated). (B) Boxplot representing the solar exposure measurements [in %], with the left boxplot representing the measurements inside the exclosure (ungrazed area) and the right boxplot representing the measurements outside the cattle exclosure (grazed area).

**Fig 3:**
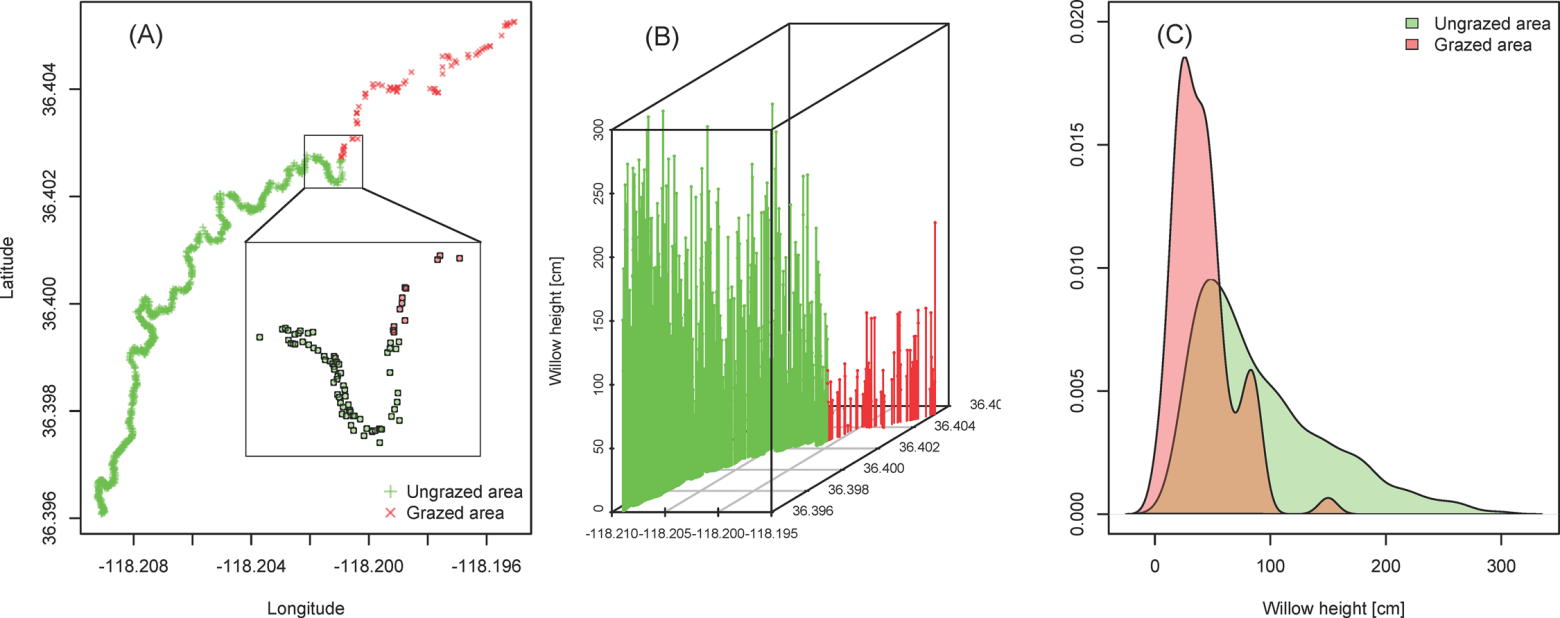
Willow height and concentration. (A) Willow location along the riverbank, with increased definition inside the inset box. (B) Individual willow heights [cm] and concentration along the riverbank with a view from the side. (C) Kernel density estimation for the willow height distribution [cm]. For each panel, green coloration represents the ungrazed area inside the cattle exclosure, and red coloration represents the area outside the exclosure in the grazed area.

In Mulkey Meadows, we found that the vegetation was not spatially structured (i.e., no spatial autocorrelation, Morans’ *I* = -0.034 ± 0.025, *p =* 0.47), but differed inside and outside the cattle exclosure. In the presence of livestock, the riparian vegetation was dominated by sedge (*Carex spp*.) while in the absence of livestock, significantly more willow (*Salix spp*.) were present (Fig 2A, chi-squared test: $\chi_{3}^{2}$ = 10.9, *p <* 0.05). Additionally, we found important differences in vegetation cover (Fig 3). In the area where cattle were excluded, we found 13 times more willows (980 trees, for a river length of 1200 m) compared to the area where cattle were present (75 trees, for a river length of 900 m). This difference can be tested with the average distance between two consecutive willows along the transect, which is 5.9 meters inside the exclosure and 12.4 meters outside (Autoregressive model: *F*_1,1049_ = 74.8, *p <* 0.001, Moran’s *I* on residuals = -0.0001 + 0.0007, *p =* 0.86). In addition, the willows in the exclosure were on average twice as tall (0.92 ± 0.56 meters) compared to the willows outside of the exclosure (0.43 ± 0.29 meters) (Fig 3C, Autoregressive model: *F*_1,1049_ = 65.3, *p <* 0.001, Moran’s *I* on residuals = -0.0012 + 0.0007, *p =* 0.10). Accordingly, the solar exposure was not spatially structured (Morans’ *I* = -0.039 ± 0.025, *p =* 0.11) and the river was shadier when cattle were excluded (84.1% sunny inside exclosure, 95.4% outside, logistic regression: *z*_28_ = 3.3, *p <* 0.05, Fig 2B). In both the grazed and the ungrazed area, we found no association between any of the water temperature metrics and the solar exposure at a given point, using both a continuous metric (percentage) and a binary metric (shade / no shade) for solar exposure (all *p >* 0.05). We found no association between cattle exclosures and river depth, water velocity, or habitat type (pool, riffle, or under bank) (all *p*-values > 0.05).

### Livestock exclusion

**Fig 4:**
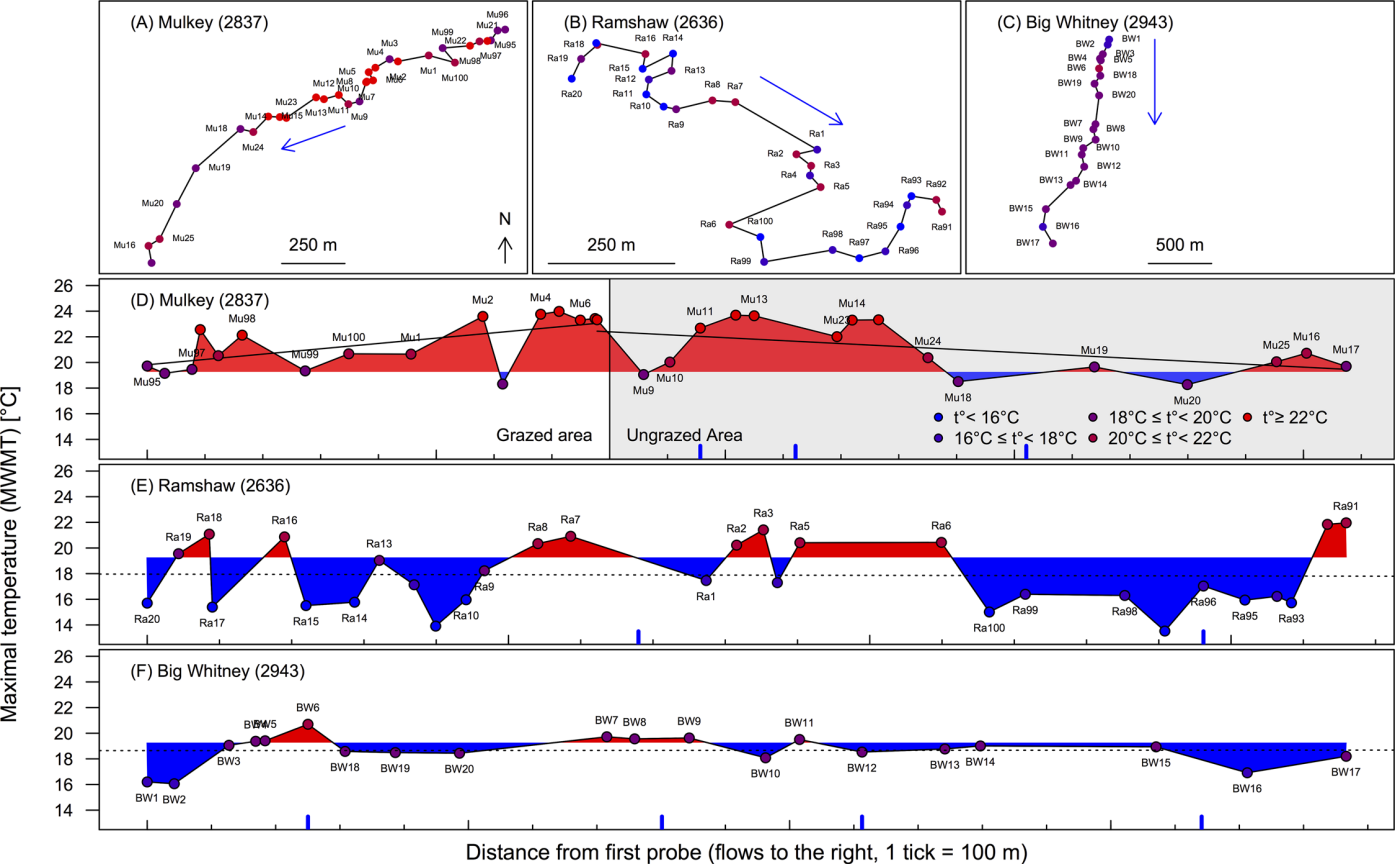
Maxima of the weekly maximal temperature (MWmaxT). The upper three panels (A-C) represent the distribution of the probes along the streambed in each meadow. All three rivers flow from North to South. The color code represents the maximum of the weekly maximal temperature (MWmaxT) in each probe: blue is used to represent probes where temperature never reaches 16°C, violet-blue when the highest temperatures were between 16–18°C, violet for 18–20°C, red-violet for 20–22°C, and red for temperatures higher than 22°C. The three larger panels (D-F) represent the same information with the maximum of the weekly maximal temperature (MWmaxT) on the y-axis and the distance from the first probe on the x-axis. The color code for the dots is the same as in the previous panels (A-C), and the red (blue) coloration between the dots represents whether the temperatures are above (below) the average weekly maximal temperature (WmaxT) observed across the three meadows. The regression lines represent the trends of maximal temperature (MWmaxT) over distance (solid lines are significant, while dashed lines are not). The grey area in Mulkey represents the cattle-exclosure, and the blue lines on the x-axis represent the different tributaries entering the system.

In Mulkey Meadows, we found increasing maximal temperatures (MWmaxT) from upstream to downstream outside the exclosure where cattle are present: 0.41 ± 0.14°C per 100 meters (Fig 4D; linear regression: *t*_14_ = 3.02, *p <* 0.01), and we did not find significant autocorrelation in the residuals (Moran’s *I* autocorrelation coefficient was equal to 0.041 ± 0.053, *p =* 0.44). The greater the distance water travelled in the stretch of stream open to cattle grazing, the warmer the stream temperatures. At the end of the cattle grazing section, in the upstream part of the exclosure, water temperatures reached 24°C each day during seven consecutive days across the study duration (MwmaxT). Interestingly, this temperature trend with distance downstream was reversed once the cattle were excluded from the river via the cattle exclusion fence: -0.25 ± 0.10°C per 100 meters (linear regression: *t*_12_ = -2.44, *p <* 0.05), again, no significant autocorrelation was found in the residuals (Moran’s *I* = 0.034 ± 0.052, *p =* 0.52). In contrast, no trends in distance were observed in maximal temperature (MWmaxT) in the two other meadows where cattle were absent since 2001 (Ramshaw, linear regression: *t*_28_ = -0.11, *p =* 0.92, Fig 4E, Big Whitney, linear regression: *t*_19_ = 0.062, *p =* 0.95, Fig 3F). We found no spatial autocorrelation in the residuals of the linear regressions in the other two meadow streams. Moran’s *I* autocorrelation coefficient was equal to 0.0006 ± 0.029 (*p =* 0.98) in Ramshaw Meadows, and 0.005 ± 0.037 (*p =* 0.90) in Big Whitney Meadow. In other words, there was a non-significant correlation between neighboring data after accounting for the distance between probes.

In Mulkey, the weekly maximal temperature (WmaxT) was on average 21.3 ± 1.9°C and the maximal value recorded (MWmaxT) was 24.0°C, which was reached on probe Mu5 (Fig 4D) just upstream of the cattle exclosure, and the minimal value recorded was 18.27°C and was reached on probe Mu20 (Fig 4D) at the end of the cattle exclosure, i.e., after the longest distance without cattle. The difference between the smallest value (the best case scenario in the ungrazed area) and the largest value (the worst case scenario in the grazed area) is therefore 5.7°C, a difference observed over a distance of 1087 meters.

### Temperature differences among meadows

**Fig 5:**
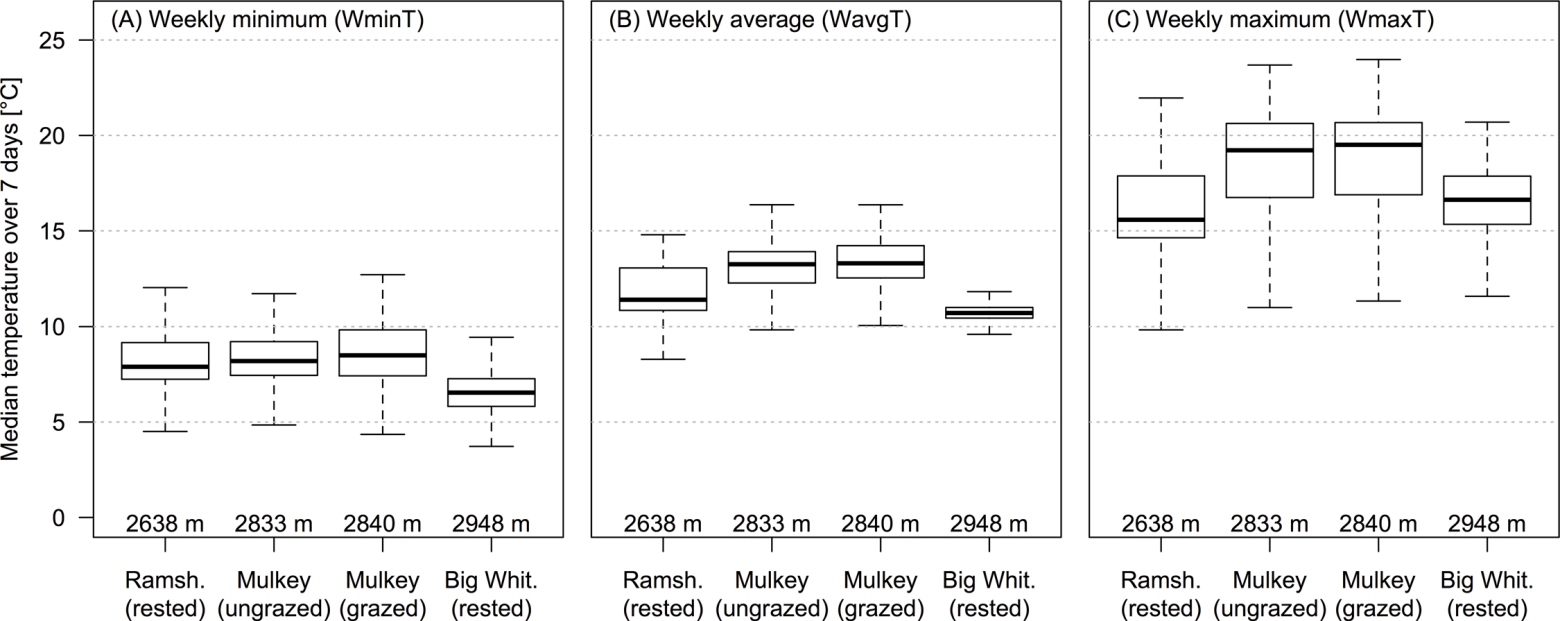
Temperature summaries. Temperature was recorded every 20–30 minutes at each probe. To limit the amount of data from each probe (and to remove temporal autocorrelation), we calculated three metrics that resulted in only one value/probe/day for analyses (See S1 Fig): daily minimum (DminT), daily average (DavgT), and daily maximum (DmaxT). To remove exceptional or erroneous data, we then computed the median value over seven day windows for each of these three metrics. Each boxplot represents the overall variation for the median daily value over seven day moving windows (WminT, WavgT, and WmaxT) in the three different meadows, with the two different areas (grazed/ungrazed) in Mulkey. The plots are arranged in order from (left to right) the lowest elevation meadow (Ramshaw) to the highest elevation meadow (Big Whitney).

The three study meadows differed in elevation with Ramshaw Meadows at 2636 m, Mulkey Meadows at 2837 m, and Big Whitney Meadow at 2943 m. Despite Mulkey’s intermediate elevation, all three weekly temperature metrics (WminT, WavgT, and WmaxT) were higher in Mulkey, which is the only meadow partially grazed by livestock in recent years (Fig 5, Table 2). Across the study duration (2010–2012), the average water temperature during the eight warmest weeks of Mulkey Meadows was 13.2°C in the grazed area and 13.0°C in the ungrazed area. In contrast, it was cooler in Ramshaw (11.8°C) and in Big Whitney (10.7°C). The monthly average median minimal temperature over 7 days (WminT) was 1.79 ± 0.24 degrees higher in the grazed area of Mulkey compared to Big Whitney (mixed model multiple regression: *t*_23_ = 7.36, *p <* 0.001), but was not significantly different from Ramshaw (mixed model multiple regression: *t*_22.9_ = 0.01, *p >* 0.05), and also not significantly different from the ungrazed part of Mulkey Meadows (mixed model multiple regression: *t*_22.9_ = 1.35, *p >* 0.05). The median average temperature over 7 days (WavgT) was on average 2.65 ± 0.27 degrees higher in the grazed area of Mulkey compared to Big Whitney (mixed model multiple regression: *t*_23.1_ = 9.65, *p <* 0.001), 1.65 ± 0.26 degrees higher in Mulkey compared to Ramshaw (mixed model multiple regression: *t*_22.9_ = 6.24, *p <* 0.001), and not significantly different from the ungrazed part of Mulkey (mixed model multiple regression: *t*_22.9_ = 1.17, *p >* 0.05). The median maximal temperature over 7 days (WmaxT) was on average 2.63 ± 0.63 degrees higher in Mulkey compared to Big Whitney (mixed model multiple regression: *t*_31_ = 4.17, *p <* 0.01), 3.53 ± 0.61 degrees higher in Mulkey compared to Ramshaw (mixed model multiple regression: *t*_31_ = 5.77, *p <* 0.001), and not significantly different from the ungrazed part of Mulkey (mixed model multiple regression: *t*_31_ = 1.17, *p >* 0.47). These results were robust with regards to the scale at which we averaged the temperatures, that is, the results were similar when we instead used yearly or weekly averages instead of monthly averages (Table 3).

### Predicted temperatures under different climate change scenarios

**Fig 6:**
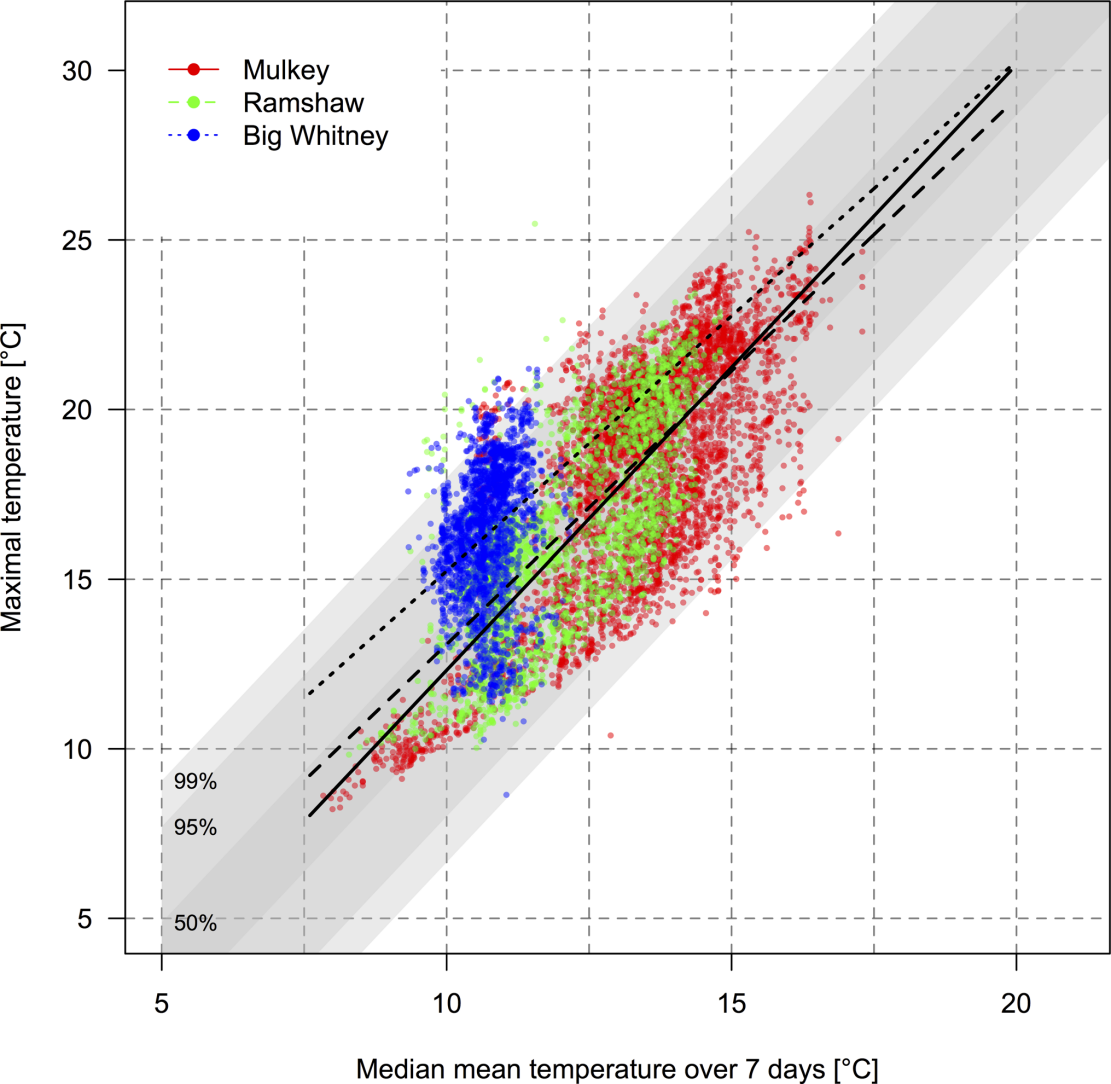
Temperature prediction model. Relationship between weekly average temperature (WavgT) [in °C] and the daily maximal temperature (DmaxT) [in °C], observed in one probe. Each point represents the temperatures from a single day measured at a single probe, red dots are for Mulkey Meadows, green dots for Ramshaw Meadows, and blue dots for Big Whitney Meadow. The three regression lines represent the three different meadows: Mulkey (solid), Ramshaw (dashed), and Big Whitney (dotted).

The relationship between the weekly average temperature (WavgT) and the maximal temperature (DmaxT), i.e., the slope of the temperature prediction model (Fig 6), is the highest in Mulkey: a one-degree elevation of the weekly average temperature is predicted to result in 1.74 ± 0.02°C increase in the maximal temperature reached. This value is 0.12 ± 0.04°C higher than in Ramshaw (Mixed model, *t*_9455_ = 2.47, *p <* 0.05) and 0.26 ± 0.10°C higher than in Big Whitney (Mixed model, *t*_9425_ = 3.18, *p <* 0.01).

**Fig 7:**
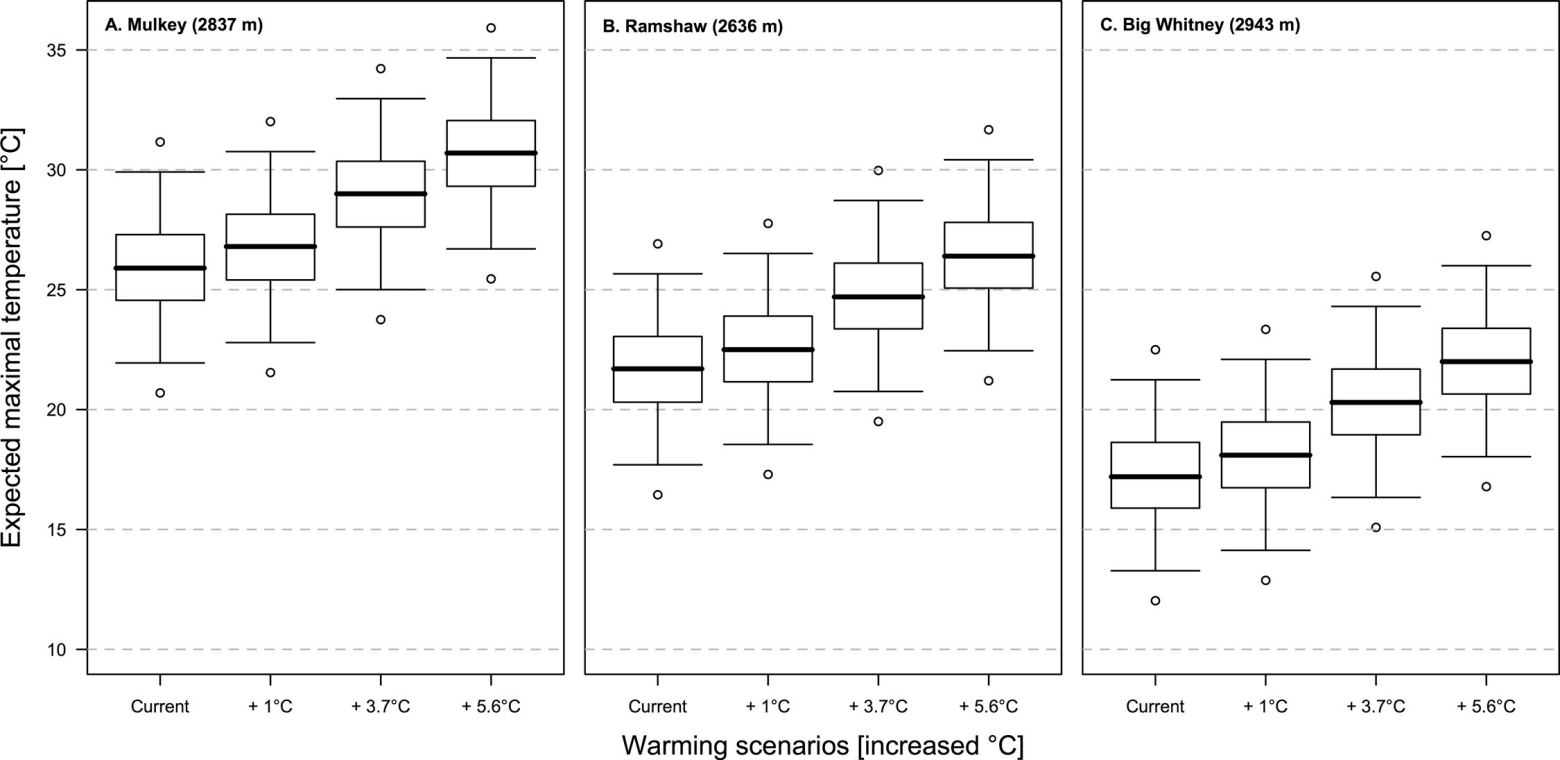
Temperature predictions under four climatic scenarios. The four climate warming scenarios represent: (1) the current situation, (2) a moderate increase of 1°C in air temperature, (3) a more realistic scenario of 3.7°C increase in air temperature, and (4) the worst-case scenario for the U.S.A., with a 5.6°C increase in air temperature. The boxplots represents, for each meadow and each scenario, the expected maximal temperature (DmaxT) (black line), the 50% prediction interval, i.e., the maximal temperatures expected every other year (box), the 95% prediction interval, i.e., the maximal temperatures (DmaxT) expected every twenty years (upper whiskers), and the 99% prediction interval, i.e., the maximal temperatures (DmaxT) expected every one hundred years (upper circle).

When we do not include warming, our model predicts that the maximal temperature (DmaxT) in Mulkey should reach on average 25.9°C, but could reach, in some parts of the river, 27.3°C every other year and 29.9°C every twenty years (Table 4 and Fig 7), which is consistent with the observed daily maximal temperature (DmaxT) that reached 26.3°C in Mulkey Meadows. In Ramshaw, the maximal temperature (DmaxT) modeled should reach 21.7°C on average, 23.1°C every other year, and 25.7°C every twenty years (Table 4, Fig 7), compared to the observed daily maximal temperature (DmaxT) which reached 25.5°C in Ramshaw meadow. Finally, in Big Whitney, the maximal temperature (DmaxT) modeled should reach 17.2°C on average, 18.6°C every other year, and 21.2°C every twenty years (Table 4, Fig 7), compared to the observed daily maximal temperature (DmaxT) of 21.2°C in Big Whitney meadow. If we consider the most optimistic scenario, i.e. a global temperature elevation of “only” 1°C by the end of the century (i.e., 0.5°C for the water temperature), our model predicts that the maximal temperature (DmaxT) reached in Mulkey could reach 28.1°C every other year in the warmest parts of the river (23.9°C in Ramshaw; 19.5°C in Big Withney) and 30.8°C every twenty years (26.5°C in Ramshaw; 22.1°C in Big Withney). With a more realistic scenario of global warming, i.e., a global temperature elevation of 3.7°C (i.e., 1.8°C in rivers), our model predicts that some parts of the river could reach 30.4°C every other year (26.1°C in Ramshaw; 21.7°C in Big Withney) and 33.0°C every twenty years (28.7°C in Ramshaw; 24.3°C in Big Withney). Modeled temperatures in the three meadows are summarized in Table 4 and Fig 7.

**Table 4:** Observed and expected (modeled) temperatures.

current	Mulkey (2837 m)	13.4	17.3	18.4	26.3	25.9	[24.6:27.3]	[21.9:29.9]	[20.7:31.2]
current	Big Whitney (2943 m)	10.7	12.2	16.5	21.2	17.2	[15.9:18.6]	[13.3:21.2]	[12.0:22.5]
1°C	Ramshaw (2636 m)	12.4	15.3	-	-	22.5	[21.2:23.9]	[18.5:26.5]	[17.3:27.8]
1°C	Mulkey (2837 m)	13.9	17.8	-	-	26.8	[25.4:28.1]	[22.8:30.8]	[21.5:32.0]
1°C	Big Whitney (2943 m)	11.2	12.7	-	-	18.1	[16.7:19.5]	[14.1:22.1]	[12.9:23.3]
3.7°C	Ramshaw (2636 m)	13.7	16.6	-	-	24.7	[23.4:26.1]	[20.8:28.7]	[19.5:30.0]
3.7°C	Mulkey (2837 m)	15.2	19.1	-	-	29.0	[27.6:30.4]	[25.0:33.0]	[23.8:34.2]
3.7°C	Big Whitney (2943 m)	12.5	14.0	-	-	20.3	[18.9:21.7]	[16.3:24.3]	[15.1:25.6]
5.6°C	Ramshaw (2636 m)	14.7	17.6	-	-	26.4	[25.1:27.8]	[22.5:30.4]	[21.2:31.7]
5.6°C	Mulkey (2837 m)	16.2	20.1	-	-	30.7	[29.3:32.1]	[26.7:34.7]	[25.5:35.9]
5.6°C	Big Whitney (2943 m)	13.5	15.0	-	-	22.0	[20.6:23.4]	[18.0:26.0]	[16.8:27.3]

## Discussion

In Mulkey Meadows, the one study meadow with a cattle exclosure, we found that riverbank vegetation was both larger and denser inside the exclosure (the ungrazed area) compared to outside the exclosure where cattle were present (Fig 3). We also found that this difference in vegetation cover was associated with more shaded waters where cattle could not reach the stream (Fig 2). Interestingly, we found an increasing pattern of maximal temperatures along the stretch of stream where cattle were present, which then reversed when cattle were excluded (Fig 4). We also found that water temperatures were cooler in the two ungrazed meadows compared to the grazed area in the partially grazed meadow (Fig 5). Finally, we found that predicted temperatures under different global warming scenarios were likely to be higher in presence of livestock (Fig 7). These results suggest that cattle in this area could impact water temperature by degrading stream vegetation, and that cattle grazing could interact with future warming and impair the resilience of these sensitive and protected ecosystems to climate change.

Active grazing has a strong effect on riparian vegetation and, under grazing, fast growing vegetation such as sedges and grasses are favored over shade-providing trees such as willows. In addition, livestock aggregate near the river to drink, and in so doing, livestock can trample and damage the riverbanks that could otherwise provide important habitat (e.g., undercut banks) for stream fishes during the warm summer season. In our study, areas without cattle tended to be covered with willow while areas that were grazed tended to be covered with sedges and grasses (Fig 2A). Moreover, the willows were much larger in areas were cattle were excluded compared to the areas where cattle grazed actively (Fig 3). Willows can provide important stream cover and, not surprisingly, stream reaches where willows were present were more shaded than reaches dominated by sedges and grasses (Fig 2B). Due to the slow recovery of these sensitive habitats [20], the difference in terms of stream shading between the two areas was relatively subtle (84% solar exposure/16% shading in the ungrazed area compared to 95% solar exposure/5% shading in the grazed area). Nevertheless this vegetation reduction and its consequences in terms of exposure to direct sunlight may be enough to explain the stream temperature differences observed between the grazed and the ungrazed area within Mulkey Meadows. Indeed, shading by riparian vegetation is known to be a major factor reducing direct heat transfer between the air and water [44–46]. For instance, deforestation has been long known to have the potential to increase warming, and even though the overall process is not completely understood [47,48], numerous studies have promoted riparian vegetation as a conservation measure to counteract the deleterious effects of temperature increases [44,45,49–52]. At our study site, we found that river temperatures were over 5°C higher where cattle were present when compared to the ungrazed area where cattle were excluded (Fig 4). Exclusion of cattle could be a relatively inexpensive, although politically challenging, measure to minimize the impact of future warming in these systems by allowing natural willow generation along the stream corridor.

Comparison among meadows showed similar temperature patterns as those described above: the water temperatures observed over the entire summer were higher in the grazed part of Mulkey compared to both ungrazed meadows, Ramshaw and Big Whitney (Fig 5). Importantly, cattle have been excluded from Ramshaw and from Big Whitney since 2001. The rested Ramshaw Meadow provides an interesting contrast to Mulkey Meadows because there are several reasons why we might expect stream temperatures to be warmer in Ramshaw than Mulkey including that (1) Ramshaw is 200 meters lower in elevation, (2) is wider but not deeper [19], and (3) has two tributary inputs compared to three in Mulkey, even though none of these tributaries seems to have a strong impact on the temperature profile (Fig 4). For all of these reasons, we expected water temperature in Ramshaw to be warmer than in Mulkey, but we observed the opposite. The observed maximal temperatures were on average 3.5°C colder in Ramshaw than in Mulkey (Fig 5, Table 3), which suggests that differences in summer temperatures between the meadows is not driven solely by the aforementioned attributes. In the future, it would be interesting to explore other metrics of temperature, such as the minimum night temperature, which might show changes in the degree of night time cooling with climate change, or the duration of warming and degree days of heat accumulated.

Increasing temperatures due to environmental change and their associated consequences are threatening biodiversity through many different processes [3,53,54] and many species are predicted to go extinct in the coming decades as a consequence [55,56]. The natural conditions of Kern Plateau open meadows combined with reduced streamside vegetation may diminish the capacity of these streams to remain cool, and future warming could result in water temperatures reaching lethal levels for the most abundant fish species in the three meadows [19], the cold-water California golden trout, as well as the aquatic invertebrate that provide a critical prey base for the fishes in these systems [57,58]. Cold-water fishes, such as salmonid fishes, are known to suffer the effects of high temperature at several different life stages [59,60]. The primary impacts include the direct effect of temperature on physiology of trout and its invertebrate prey, and the reduced concentration of dissolved oxygen in warmer water [3]. Another threat of warmer air temperatures includes increased rainfall in high elevation areas [61], which can alter flow regimes with consequences for early life survival [62,63]. Higher water temperatures are also expected to trigger earlier spawning at smaller sizes [64], which could potentially affect juvenile survival [65]. Finally, parasites and disease are more prevalent in warmer water and are known to increase mortality in wild salmonid populations [66,67]. Of course, it is important to recognize that increasing temperature could also have positive effects, including a longer growing season and potentially higher over-winter survival [68].

Modeling the expected increase in temperature given a warming scenario in air temperature is not straightforward [41,69] because the relationship between air and water temperatures is not linear [42,69,70], and the magnitude depends on several factors. For example, it has been shown that local and climatic factors may have a strong influence on the maxima, whereas other metrics, such as minima or mean, might be more influenced by landscape factors [46]. Data from 43 rivers across the globe has shown that the average ratio of water and air temperature increases was between 0.6 and 0.8, with several streams showing higher ratios [42]. Some models suggest a 2.4°C to 4.7°C increase in water temperature across the USA if the CO_2_ concentration doubles, and in non-shaded areas, this increase could rise to an additional 6°C during summer [71]. We used a very conservative estimate for the expected increased water temperature of 0.5°C per increased degree in air temperature. This value is conservative in that it is below the lower bound proposed by Morrill *et al*. [42] and because expected maximal (minimal) temperatures are likely to be underestimated (overestimated) by models [41,70].

While our study focused on the effects of grazing on stream temperatures and while we found support for the hypothesis that grazed meadows tend to be warmer than ungrazed meadows, we cannot rule out other possibilities. For example, tributary inputs could influence overall temperatures among the three meadow systems. In our case, the three systems had comparable numbers of tributary inputs (2 in Mulkey, 3 in Ramshaw, 4 in Big Whitney), and a fine-scale examination of the stream temperature data from probes in the vicinity of these inputs suggest only a localized effect that could result in either warming or cooling depending on the particular tributary. Another factor that could play a role is aspect, but both Mulkey (grazed) and Ramshaw (ungrazed) are south facing, although Ramshaw flows southeast, while Mulkey flows southwest (Fig 1). Watershed area, watershed elevation, and flow velocity could also play a role and Ramshaw has a smaller watershed area located at a lower elevation, making predictions challenging (Table 1). Flow velocity is not significantly different between the Ramshaw and the grazed part of Mulkey (ANOVA, *F*_1,38_ = 2.35, *p >* 0.05). Finally, differences in the magnitude of groundwater inputs could be playing a role. Unfortunately, we do not have a handle on the extent that groundwater inputs differ among the three study meadows. Other weaknesses of the study include a lack of air temperature data specific to each meadow, a lack of temperature data prior to the construction of the cattle exclusion in the rested meadows which precludes a before-after comparison, lack of data on stream temperatures in the area below the cattle exclusion in Mulkey, and the low replication overall. Moreover, comparisons to pristine meadows that have never been grazed would have been ideal, but none exist in our study region.

Looking to the future, our temperature modeling suggests that temperatures—under all warming scenarios—are predicted to be much higher in Mulkey Meadows, where cattle are present, than in the other two meadows where cattle have been excluded (Fig 7, Table 4). Moreover, the slope of the relationship between the weekly average temperature (WavgT) and the maximal temperature reached (DmaxT) was steepest in Mulkey Meadows, which indicates a potential interaction between climate warming and grazing (Fig 6)–i.e., intensified warming in the presence of cattle. Even with a relatively optimistic scenario, by the end of the 21^st^ century, water temperatures exceeding 30°C will be frequently reached in the partially grazed Mulkey Meadows. Prolonged time at temperatures above 25–26°C are known to be lethal for some subspecies of rainbow trout [72] and temperatures above 30°C are lethal for most salmonids [73]. Rainbow trout (*Oncorhynchus mykiss*) in southern California occur in streams with temperatures up to 28°C, but fish in these systems are known to avoid the warmer areas of the rivers by seeking out cool water seeps [74]. Little is known about the heat tolerance of the California golden trout, in particular the sublethal effects of temperature on growth and reproduction, and it is possible that these fish could persist in thermal refuges created by groundwater inputs or stratified pools when temperatures rise, but the absence of information on the extent of refuge habitat in these meadows combined with the likely increase in water temperature in the coming decades suggests that a precautionary approach is warranted. Meadows in the Sierra Nevada have already experienced widespread degradation from overgrazing in the late 1800s and early 1900s, and need many years to recover once degraded [20]. Since global climate change is likely to continue due to the inertia of climate [1], management strategies removing additional stressors might be necessary to protect freshwater ecosystem integrity and biota [3].

Increased water temperatures associated with cattle grazing may not only harm fish populations through testing their thermal limits, but cattle grazing is also likely to degrade the montane meadows through erosion and xerification [23]. Cattle grazing has been demonstrated to modify entire meadow ecosystems, and small scale-cattle exclosures have shown poor restoration potential compared to large-scale cattle removal [21]. For these reasons, livestock grazing and associated effects are recognized as a long-term stressor known to impair the resilience of public lands to the impacts of climate change [16,21]. Indeed, Beschta *et al*. 2013 advocate for a careful documentation of the ecological, social, and economic costs of livestock on public lands, and suggest that costs are likely to exceed benefits in these sensitive ecosystems. Overall, our results provide further support that land use can interact with climate change to intensify warming in high elevation meadow ecosystems. In sensitive systems such as these, restoration measures could be taken to reduce the management stressors that accentuate the impacts of climate change [16,75–77].

## Supporting Information

S1 Fig Temperature metrics. Example of individual measurements (black dots) in one individual location (Mu8 in Mulkey Meadows). Red dots (respectively violet and blue), represent the daily maximal temperature value (DmaxT) (resp. mean and minimal). The solid lines represent the moving median over seven days (WmaxT, WavgT, WminT) and the circled values represent the maximal values of the moving averages, i.e., the MWmaxT (red), and the MWavgT (violet). The circled dot (red), represents the true maximal value observed.
(PDF)
