# Supplementary material for: Mediating Water Temperature Increases Due to Livestock and Global Change in High Elevation Meadow Streams of the Golden Trout Wilderness
Source: PLoS One. 2015 Nov 13;10(11):e0142426. doi: 10.1371/journal.pone.0142426 (PMC4643935; doi:10.1371/journal.pone.0142426)
Supplement: S1 Fig — Example of individual measurements (black dots) in one individual location (Mu8 in Mulkey Meadows). Red dots (respectively violet and blue), represent the daily maximal temperature value (DmaxT) (resp. mean and minimal). The solid lines represent the moving median over seven days (WmaxT, WavgT, WminT) and the circled values represent the maximal values of the moving averages, i.e., the MWmaxT (red), and the MWavgT (violet). The circled dot (red), represents the true maximal value observed. (PDF) [file pone.0142426.s001.pdf]

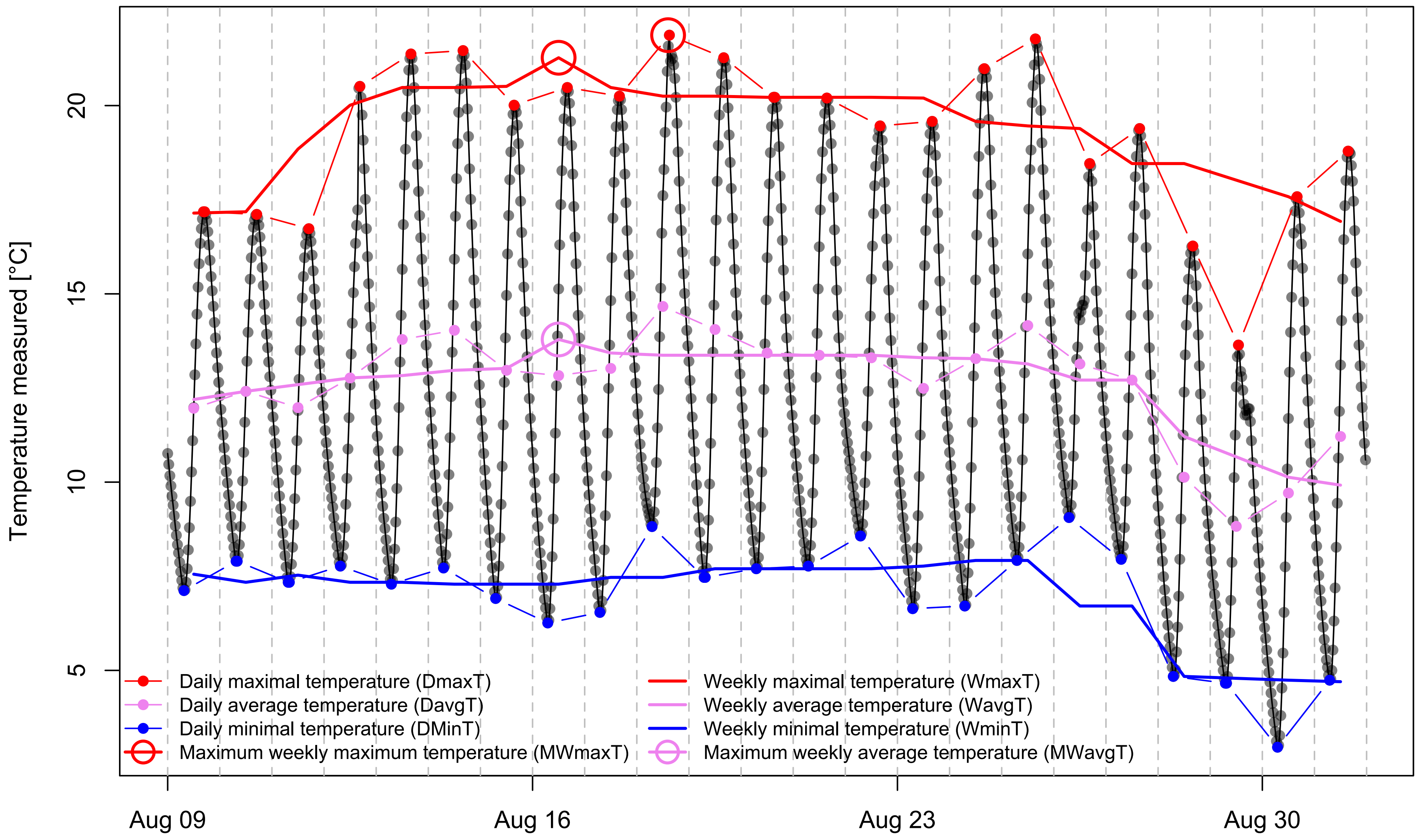

Supplementary Figure 1. Example of individual measurements (black dots) in one individual location (Mu8 in Mulkey Meadows). Red dots (respectively violet and blue), represent the daily maximal temperature value (DmaxT) (resp. mean and minimal). The solid lines represent the moving median over seven days (WmaxT, WavgT, WminT) and the circled values represent the maximal values of the moving averages, i.e., the MWmaxT (red), and the MWavgT (violet). The circled dot (red), represents the true maximal value observed.
